# Online reach and engagement of a child nutrition peer-education program (PICNIC): insights from social media and web analytics

**DOI:** 10.1186/s12889-022-13252-3

**Published:** 2022-04-26

**Authors:** Maria Henström, Kerith Duncanson, Clare E. Collins, Lee M. Ashton, Emma Davidson, Richard Ball

**Affiliations:** 1 Department of Biosciences and Nutrition, Karolinska Institutet, 141 83 Huddinge, Sweden; 2 School of Health Sciences, College of Health, Medicine and Wellbeing, University of Newcastle, Callaghan, NSW 2308 Australia; 3 School of Medicine and Public Health, College of Health, Medicine and Wellbeing, University of Newcastle, Callaghan, NSW 2308 Australia; 4 Priority Research Centre for Physical Activity and Nutrition, University of Newcastle, Callaghan, NSW 2308 Australia; 5 School of Education, College of Human and Social Futures, University of Newcastle, Callaghan, NSW 2308 Australia; 6 Hunter New England Local Health District, Wallsend, NSW 2287 Australia; 7 Mid North Coast Local Health District, Port Macquarie, NSW 2444 Australia

**Keywords:** Social media, Child nutrition, Peer education, Child feeding practices, Parents, eHealth intervention, Website, Google analytics

## Abstract

**Background:**

Parents frequently seek parental advice online and on social media; thus, these channels should be better utilized in child health interventions. The Parents in Child Nutrition Informing Community (PICNIC) program aims to facilitate peer-to-peer sharing of evidence-based child feeding information and support parents within their social networks. The present study aimed to explore web and social media analytics to evaluate reach and user engagement with the PICNIC online components.

**Methods:**

Online user activity data from the PICNIC Facebook closed group and public Page were collected through Facebook Insights, and program-specific website traffic data through Google Analytics. Analytics data from Nov-2019 to April-2021 was evaluated through visualisation and summary statistics to obtain insights into program growth and current reach in Australia, compare demographics of audience reached through the online channels, and explore parents’ use and engagement in PICNIC content.

**Results:**

Results showed steady program growth in the 18 months of recruitment; participant numbers grew from 102 to 261 peer educators while the Facebook Page audience increased threefold, totalling 1615 followers. Intervention posts shared on Facebook (4–5 posts/week) typically reached only a portion of PICNIC Page followers each week, but also reached a wider audience through their friends. Throughout the evaluated period, Facebook users actively engaged in PICNIC posts, although the level of engagement varied considerably from post to post. Furthermore, results from this study suggest the strategy of directing potentially interested parents from social media to the website for program sign-up was successful. Finally, the explored data gave insights into users’ availability, demographics and engagement, which will be used to inform refinement of the PICNIC website and social media strategies.

**Conclusions:**

Our findings confirm the benefits of using a peer education approach and existing social network channels to disseminate evidence-based child feeding information to parents. This study also demonstrates the usefulness of web and social media analytics to be used as part of a continuous evaluation for gaining insight to inform further development and improvement of program strategies.

**Trial registration:**

The PICNIC project was retrospectively submitted for registration with the Australian New Zealand Clinical Trials Registry (ANZCTR), ACTRN12622000230752 (09/02/2022).

**Supplementary Information:**

The online version contains supplementary material available at 10.1186/s12889-022-13252-3.

## Background

Early prevention is of paramount importance in order to counteract the increasing prevalence of overweight, obesity and lifestyle related diseases worldwide [1, 2]. Eating behaviour and dietary patterns develop early in life [3], and often track into adulthood where unhealthy habits are difficult to change [4–6]. Children’s eating behaviour is largely influenced by parental food habits and feeding strategies [7, 8]; therefore, parents are an important target group in preventive health interventions. Parents believe food is crucial for their child’s health, but also describe child feeding issues as stressful and challenging [9–11]. Qualitative research further shows parents’ child feeding behaviours are influenced more by other parents than by dietary guidelines [11], and first-time parents particularly express a great need of peer support [10]. Moreover, increasing evidence describes how today’s parents often turn to the Internet and online social networks to seek parental and child health information [12–14].

Parents in Child Nutrition Informing Community (PICNIC) is a peer education program aimed at supporting parents of infants and toddlers to help improve child feeding practice and diet quality [15]. Parents are trained in Dietitian-led workshops, provided with evidence-based child nutrition and feeding information to share within their social networks. PICNIC is an implementation project which has evolved into its current format over the past 7 years [16]. The PICNIC peer nutrition education model was informed by formative research [11, 17, 18] and a pilot study [19]. Recommendations from the pilot study participants (*n* = 28) included improved access to online information by using social media platforms and website, and messages more focused on child feeding practices and appealing to parents in order to reach and positively influence behaviour. Providing a project-specific website, a closed Facebook (Fb) group and public social media pages, the PICNIC program is designed to meet parents ‘where they are’, and is expected to facilitate evidence-based information dissemination to reach and influence parents also outside the study population [15, 16].

Social media is a rapidly emerging research topic, both in terms of understanding harms and spread of misinformation, but also the potential to positively influence people’s health and well-being [20–24]. Health promotion programs delivered via online networking platforms may be effective in reaching and influencing a large population [25], in particular parents who increasingly use social media to seek parenting and health advice [14]. Consequently, social media is more widely used in parent directed child health interventions [26–32]. However, health professionals and researchers can have difficulty getting traction online, creating credible online ‘go-to’ information sources for parents, within the overwhelming amount of information (from trustworthy to highly detrimental) parents are exposed to online. Social media algorithms determine which posts are shown to whom in their News Feed, presenting challenges when trying to disseminate content, especially organic (non-paid), through these platforms [33, 34]. Therefore, online interventions need to be co-developed with end-users and evaluated continuously (both subjectively and objectively) to understand program success and identify opportunities for improvement [35–37].

Businesses frequently use web and social media analytics and tools to optimise website and digital marketing performance, and this has potential to be applied in research. Objectively collected user activity and engagement data may provide useful insight into the reach and impact of an intervention, contribute to improvement of content- and online strategies, as well as guide allocation of resources and efforts. Although analytics is sometimes used to evaluate online interventions, the selection and processing of data varies greatly between studies, and there is still little guidance available on how to use web and social media analytics as part of process evaluations.

Participatory Action Research (PAR) enables PICNIC project participants to shape and improve the program through their engagement and feedback [16, 38]. In addition to focus group discussions and correspondence with parents, online resource usage is measured continuously using standard analytic tools. Monthly dashboard reports provide the research team with key metrics from website and social media for process evaluation. This study further explored and visualised web and social media analytics to evaluate reach and user engagement with the PICNIC online components. Specifically, the aims were to:

- Explore and describe the growth and current reach of the PICNIC model to parents through the online platforms (project website and Facebook).
- Describe demographics of the audience reached through the online dissemination methods in Australia and compare them to the enrolled parents trained in the program.
- Explore the audience’s use and engagement in the PICNIC online intervention content.

This study will inform ongoing implementation and content refinement of the PICNIC program including social media strategies, and improved measurement of online data for future evaluations. In addition, this paper will provide guidance for health professionals/researchers to develop and/or evaluate existing or future eHealth interventions.

## Methods

### PICNIC website and social media components

**Fig. 1:**
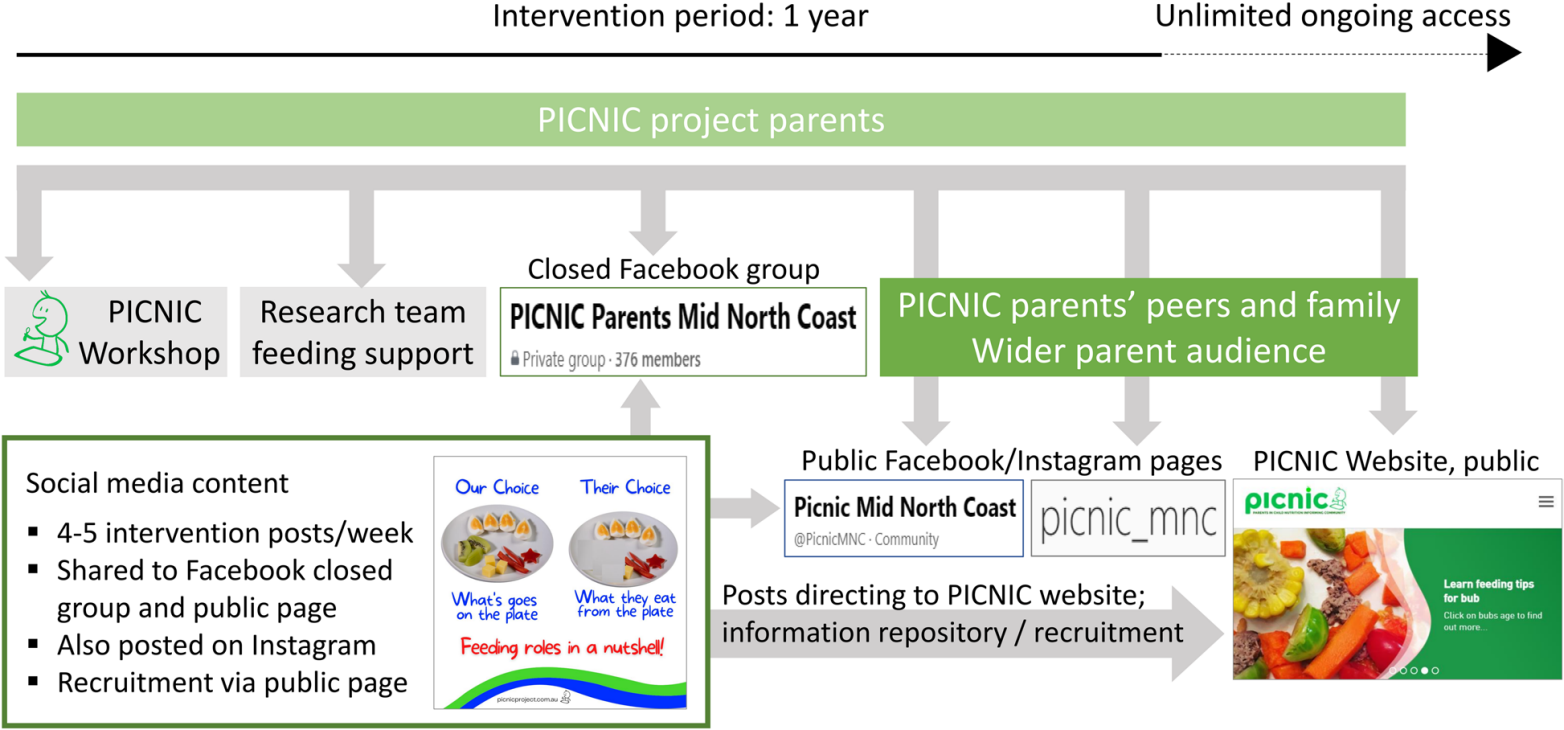
PICNIC online components. Overview of child nutrition and feeding resources provided through different channels in the PICNIC program

The PICNIC project study protocol provides a detailed description of the intervention and research methods [15]. In brief, enrolled parents engage in an introductory two-hour workshop led by an Accredited Practising Dietitian, and are then provided with child nutrition and feeding information and support through online resources for at least a year (Fig. 1). The freely accessible project-specific website (picnicproject.com.au) acts as an information repository and platform for recruitment. PICNIC parents (‘peer educators’) are invited to a closed Group on Fb, which provides continued education, support and an opportunity to connect. Originally, this forum was created and housed on a password protected portal within the website, but was moved to Fb in Jul-18 because of feedback from parents who specifically asked to receive information through channels they already use [16]. Parents are encouraged to like/follow the Fb Page “Picnic Mid North Coast” and Instagram account “picnic_mnc”, where child feeding messages are regularly (4–5 times/week) shared by the research team as ‘posts’. The posts sometimes refer to the website for more information on the topic, or how to enrol in the program. While the closed Fb group was developed to facilitate communication between enrolled parents and the research team, the Fb Page is open to the public and used as a channel to share and disseminate evidence-based information to parents’ networks and the broader online community, with the aim to further reach parents of children 0–3 year who are not (yet) enrolled in the program. All intervention messages (‘posts’) are posted by the PICNIC research team both in the closed group and on the public Page simultaneously, since content in closed groups cannot be shared publicly on Fb. Moreover, enrolled PICNIC parents are invited (through the closed Fb group) monthly to online follow-up sessions to further discuss feeding experiences with each other and the Dietician. The level of support is individual; parents choose themselves how much they want to engage in the program and can also reach out to the research team with specific questions when needed. Although PICNIC is designed to provide support for 1 year, parents are welcome to continue to engage in the program also after the intervention period.

### Study overview: process evaluation using analytics

This study uses PICNIC website and social media analytics to describe and evaluate online components of the PICNIC program. This evaluation is an important aspect of the PICNIC PAR process, facilitating adjustment of the social media content for optimal alignment with end-users’ needs [16]. According to the newly described CSD-IES social marketing planning framework, the evaluation stage is important for understanding reach and efficiency of an implemented program, and should be used as part of an iterative process towards sustainability and behaviour change [35]. In the present study we focused on the growth and reach of the program online (i.e., number of people that encounter the intervention on social media and the website; who; when; and how), as well as their interaction and engagement (i.e., post likes/shares/comments/clicks, website page views etc.) with the different parts of the online resources. Contextual factors included: ongoing recruitment with regular workshops held, the target audience (parents of young children residing in Australia), seasonal changes and the Covid-19 pandemic. When social distancing rules were introduced in late Mar-20, PICNIC went from face-to-face workshops to fully online*.*

**Fig. 2:**
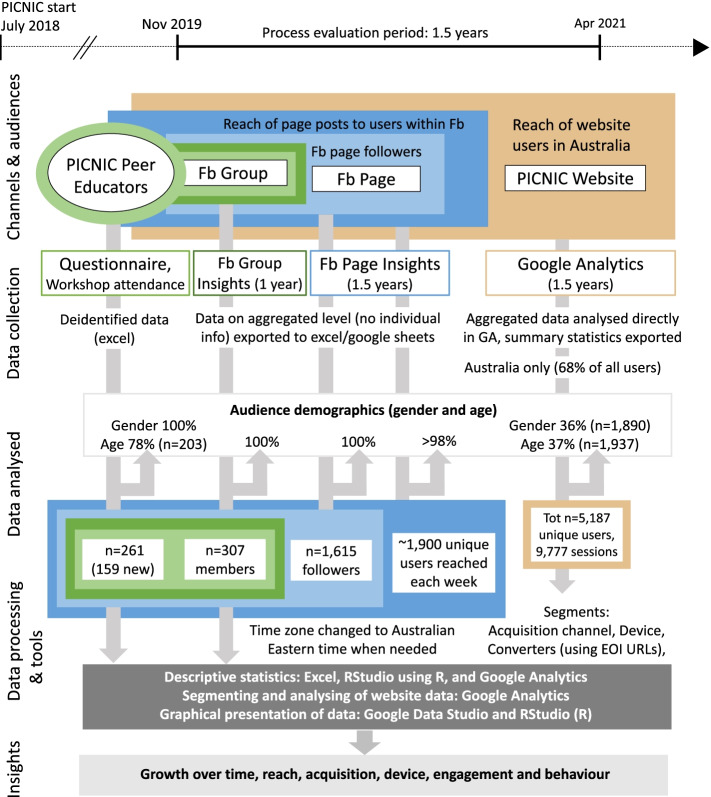
Study overview. Sources of data collected, audiences and type of data evaluated, data processing, tools used and information/insights gained. Fb, Facebook; GA, Google Analytics; EOI; Expression of Interest

Figure 2 provides a study overview. Since Fb has been the primary social platform used by PICNIC parents so far (~ 4 times more followers than on Instagram), we focused our analysis on Fb and the website. Evaluated audiences include: enrolled peer educators; Fb Group members; Fb Page followers; Fb users reached by PICNIC Page content/posts; and website visitors within Australia. Fb Group members were peer educators, but also PICNIC health professional staff/researchers and engaged parents who had not yet attended a PICNIC workshop. The PICNIC Fb Page was first created in 2014 to be used in the pilot study [19]. Four years later, recruitment for the PICNIC study [15] was initiated, although more intense recruitment took place from the end of 2019, after a series of changes had been made due to feedback (incl. Website development). For the purpose of this study, a 1.5-year evaluation period (Nov-19 – Apr-21) was selected. This covers the most intense recruitment period so far. All metrics/dimensions and analytics terms relevant to this study are fully explained in Additional file 1.

### Data collection and description of measures

#### Enrolled peer educators in the PICNIC program

The PICNIC program is directed to parents of infants or toddlers aged 0–3 years. In 2019, 2207 women gave birth in the Mid North Coast (2.4% of all births in New South Wales [NSW]) [39], where PICNIC recruitment so far has been conducted. This results in an estimated target population of approximately 4400 new parents every year (assuming each child has two parents). For the purpose of the present study, previously collected demographic data (gender and age) on peer educators enrolled in the program was included, as well as the date when the participant attended the workshop (considered the date of enrolment).

#### Facebook group and page data – Facebook insights

Administrators of Fb Groups can access and export group data through the tool *Group Insights*, with data available for up to 1 year retrospectively (some metrics stored for only 28 days). For the purpose of the present study, the Fb group analytics data used included; total number of group members, their demographics (aggregated data on gender and age), and daily number of active members (i.e., who viewed, posted, or commented/reacted to group content) [see Additional file 1]. Individual group members’ post content or comments were not assessed.

Considerably more analytics data is available for Fb Pages. The data is viewed and exported (max 2 years) in *Page Insights*, and aggregated on a group level with no link to specific individuals. By using a customised layout, relevant page- and post-level metrics [see Additional file 1] were exported to Excel sheets for the 1.5-year evaluation period. Data included daily and weekly key metrics describing growth (Page ‘fans’/followers), organic/paid reach (number of unique users who saw content) and engagement (likes/reactions, comments, shares and consumptions/clicks). Non-viral reach was used to assess the organic reach within the audience connected to the Page, that is, to what extent Page posts/content are shown by Fb to the Page fans/followers. Viral reach was explored to understand the ‘peer-to-peer’ organic reach, i.e., people reached by PICNIC posts because of their friends’ engagement. Moreover, engagement rate was calculated as Lifetime Engaged Users (Unique Users) / Lifetime Post Total reach (Unique Users)*100, and used as an estimate of how many people of those viewing a post, also chose to engage with it [40]. Finally, detailed metrics in separate spreadsheets were included, such as aggregated data on Page followers stratified by age and gender, and hourly data on the number of Page fans online on Fb each day (to understand audience availability online).

#### Website data – Google analytics

Google Analytics (GA) [41] is a web analytics service that tracks (using JavaScript codes installed on web pages) and reports website traffic, performance and user insights [42]. GA is widely used by businesses and digital marketers to test and improve marketing campaigns, understand customer behaviour, and to optimise and drive more traffic to the website. In PICNIC, we used GA Universal (administered by Go4 Multimedia) with one unfiltered view installed and data reported in Australian Eastern time zone (AEST/AEDT). The *Audience*, *Acquisition* and *Behaviour* reports in GA were explored for the specified time period, together with customised segments and primary/secondary dimensions to obtain insights. Gender and age distribution was summarised for the subset of website users who had accessible demographics data [see Additional file 2]. To assess popular pages within the website, Unique Page View metrics were plotted in a heat map. Furthermore, search terms were extracted from the *Behaviour* report using the specific URLs for search results pages. Finally, ‘conversions’ were explored by comparing overall acquisition and behaviour metrics between visits with and without submission of the Expression of Interest (EOI) form. A full list of metrics obtained from GA is available in Additional file 1.

### Data processing and statistical analysis

An overview of the study process is provided in Fig. 2. Since the intervention was based in NSW, with parents recruited locally, the present process evaluation focused on the Australian population. The vast majority of PICNIC Fb Page followers (97%) were in Australia, and as filtering for country was not possible in Fb Insights, unfiltered Fb data was used. However, 32% of the PICNIC website users were situated outside Australia; therefore, website traffic data was segmented and described for Australian users only. Data from Fb and peer educators were checked for any missing values, explored (partly through visualisation) and summarised. The website data was analysed directly in GA, or linked to Google Data Studio (GDS); a free interactive visualisation tool [43]. Descriptive statistics were provided in GA, or produced in Excel and RStudio using R version 4.1.0 (2021-05-18). Typically, normally distributed data was reported as ‘mean (standard deviation [SD])’ and data that was not normally distributed reported as ‘median’. Plotting of data was performed in GDS and heat maps were created in RStudio using R and the ggplot2 package. Detailed explanations of relevant terms and dimensions/metrics used in this study are available in the table in Additional file 1, together with comments on their use, and some considerations regarding interpretation. In Additional file 2 we also provide more details on the specific analyses performed in this study.

## Results

### Growth of the PICNIC online community

**Fig. 3:**
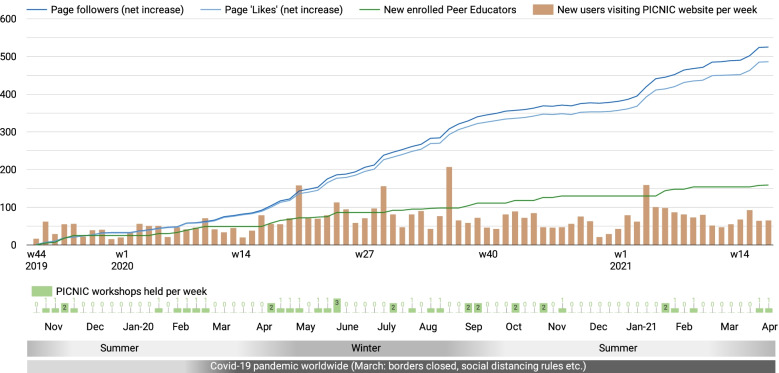
Growth of the PICNIC program online. Time chart for the 1.5-year evaluation period, comparing new peer-educators enrolled in the program, with the net increase of Fb Page fans (Page ‘likes’) and followers, as well as weekly number of new (first-time) website users in Australia. Contextual factors shown (bottom): peer educator workshops held, months/seasons, and the Covid-19 pandemic

One hundred and fifty-nine new parents enrolled as PICNIC peer educators in the 1.5-year evaluation period (Nov-19 to Apr-21), and 102 were existing PICNIC peer educators who had been trained since the initiation of the program (Jul-18). All 261 peer educators were invited to the closed Fb group for continued support. This group, which also included PICNIC health professional staff and some PICNIC parents not yet enrolled, had 307 members at the end of the observation period. PICNIC peer educators were also encouraged to like and follow the public Fb Page “Picnic Mid North Coast”. Fig. 3 shows the net increase of Page ‘fans’ (i.e., likes) and followers (typically people both like and follow a Page). The Fb Page audience grew steadily by 48% over the 1.5 years from 1090 to 1615 total followers. This means, for each new parent enrolled in the program the Fb Page audience increased by more than three followers. Meanwhile, the PICNIC website had regular traffic, with an average of 65 (SD 33) new users per week in Australia (see bars in Fig. 3).

### Reach of the intervention content

**Fig. 4:**
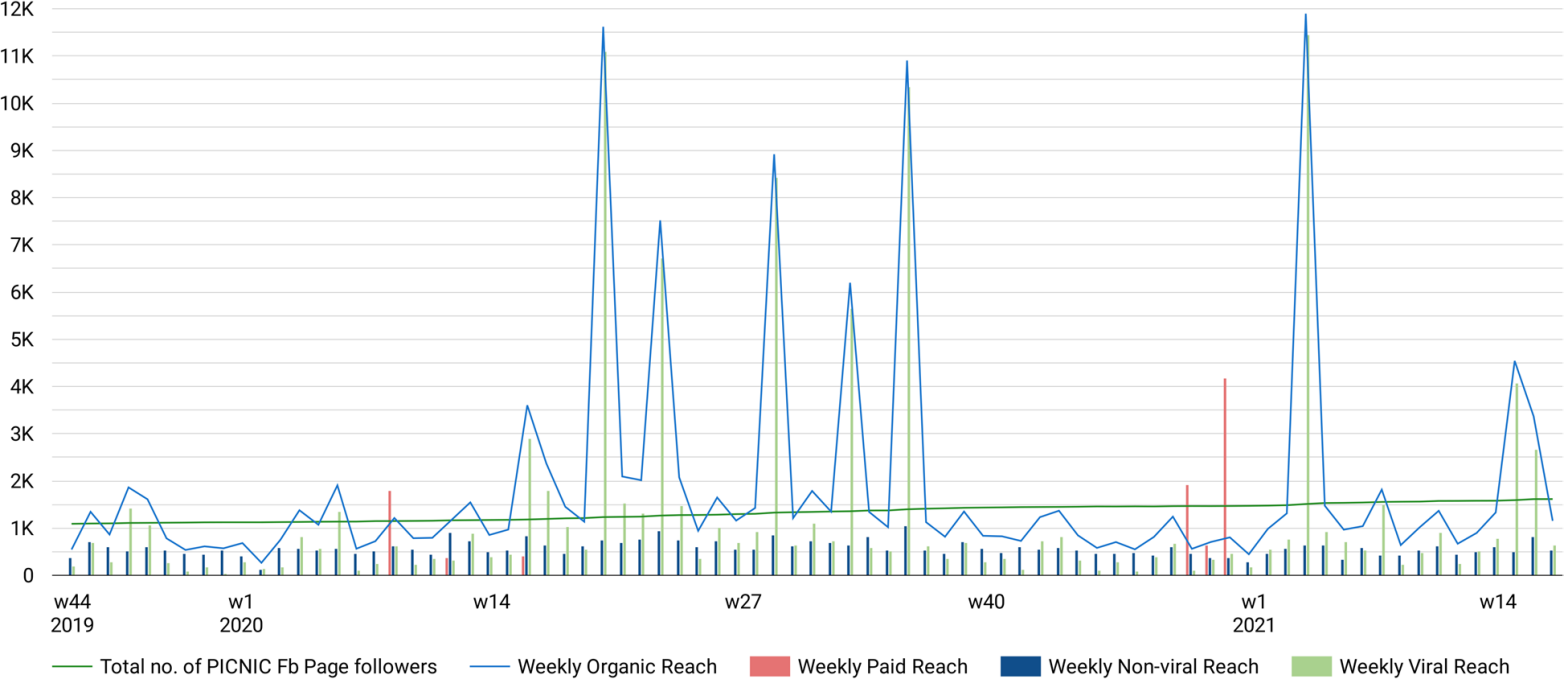
Reach on Facebook. Weekly reach of the PICNIC Fb Page content in relation to the total number of Page followers over time. Organic Reach: the weekly number of unique users who saw any Page content (typically posts) without any paid distribution. Paid reach: the weekly number of unique users who saw Page content through paid distribution (in this case, ‘boosted’ posts). Non-viral Reach: weekly organic reach to people connected to the Page (such as Page followers or fans). Viral Reach: weekly organic reach to people who saw Page content because of their friends (who for instance liked the Page, or shared or engaged with a PICNIC Page post). Fb, Facebook; w, week of the year

During the 1.5-year period a total of 359 posts (4–5 posts/week) were posted by PICNIC administrators on the Fb Page. All were organic posts, although five were then ‘boosted’ (paid). Figure 4 shows weekly reach metrics, demonstrating the overall capacity of PICNIC Page content to reach Fb users. Total organic reach varied greatly from week to week, with Page content (i.e., primarily posts) shown to between 265 and 11,895 unique users each week (median 1136 users/week). On a ‘per-post’ level, the number of people who were exposed to individual posts also varied greatly but were most often around 400 (median organic reach 398.0 users/post). Impressions of posts i.e., the total number of times a post was shown to someone, was on average 738.5 impressions/post (median 459.0). This suggests that people who were reached by a particular post typically saw that post only once. The most disseminated posts (reaching more than 3000 people through viral reach; see the ‘spikes’ in Fig. 4) were primarily recruitment posts (*n* = 5) prompting users to visit the PICNIC website for more information on the program. Some were also intervention posts (*n* = 2) with child feeding messages.

Moreover, by comparing the non-viral reach (direct reach to people connected to the Fb Page, such as fans or followers) with the number of Page followers continuously over the entire period (Fig. 4), none of the posts appeared to reach all followers, and the non-viral reach percentage was estimated at 15% if calculated daily, and 44% on a weekly basis. This means that typically only a portion of Page fans/followers were reached by the posts. Similar calculations using non-viral reach data for each specific post also confirmed this; posts reached on average one in four (24.6%) people directly connected to the Page. Furthermore, Fig. 4 also demonstrates considerable viral reach (the ‘peer-to-peer’ type of organic reach), especially during specific weeks, with between 37 and 11,450 people reached per week through their Fb friends (median viral reach 585 people/week). This indicates that Page content was spread to a wider audience than only those following the Page.

**Fig. 5:**
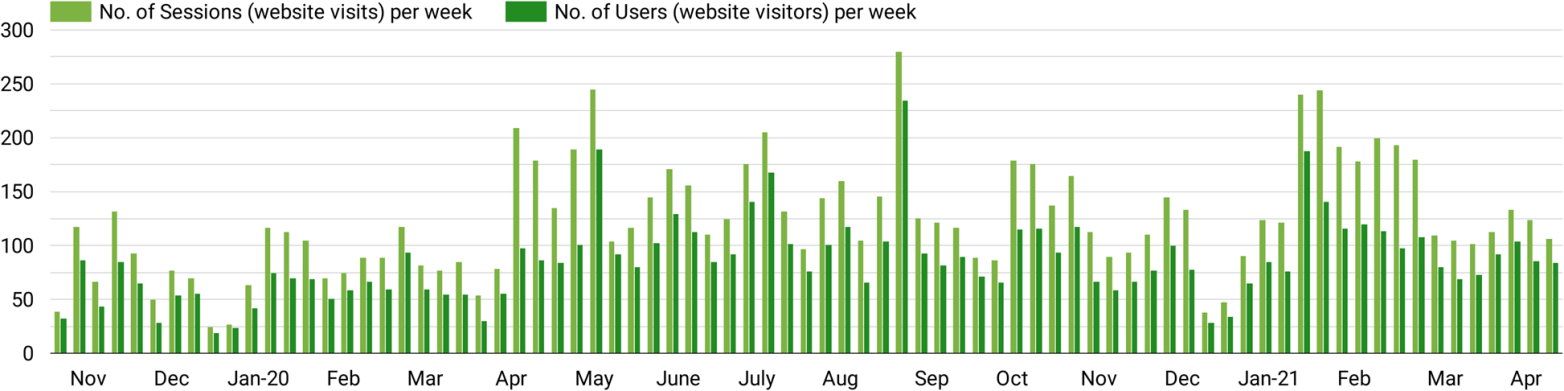
PICNIC website reach. Weekly number of sessions and unique users visiting the website

The PICNIC website was frequently visited during the whole 1.5-year period with a total of 9777 website visits (sessions) from 5187 unique users within Australia. The website reach is visualised in Fig. 5, with ‘traffic’ data showing the number of unique sessions and users per week. Each week, the website was visited on average 124 times (SD 53) by 86 (SD 38) unique users in Australia. The plotted data illustrates lower website activity during December and January when Australian summer holiday occurs, and recruitment of peer educators was not as intense. The worldwide Covid-19 pandemic began to substantially limit social and physical interaction in Australia in Mar-20. PICNIC analytic data indicates an upturn in online intervention reach around this time, with increased website usage after Apr-20 (averaging 613 sessions/month as compared to 404 sessions/month during Nov-19 to Mar-20).

### When the audience is online

**Fig. 6:**
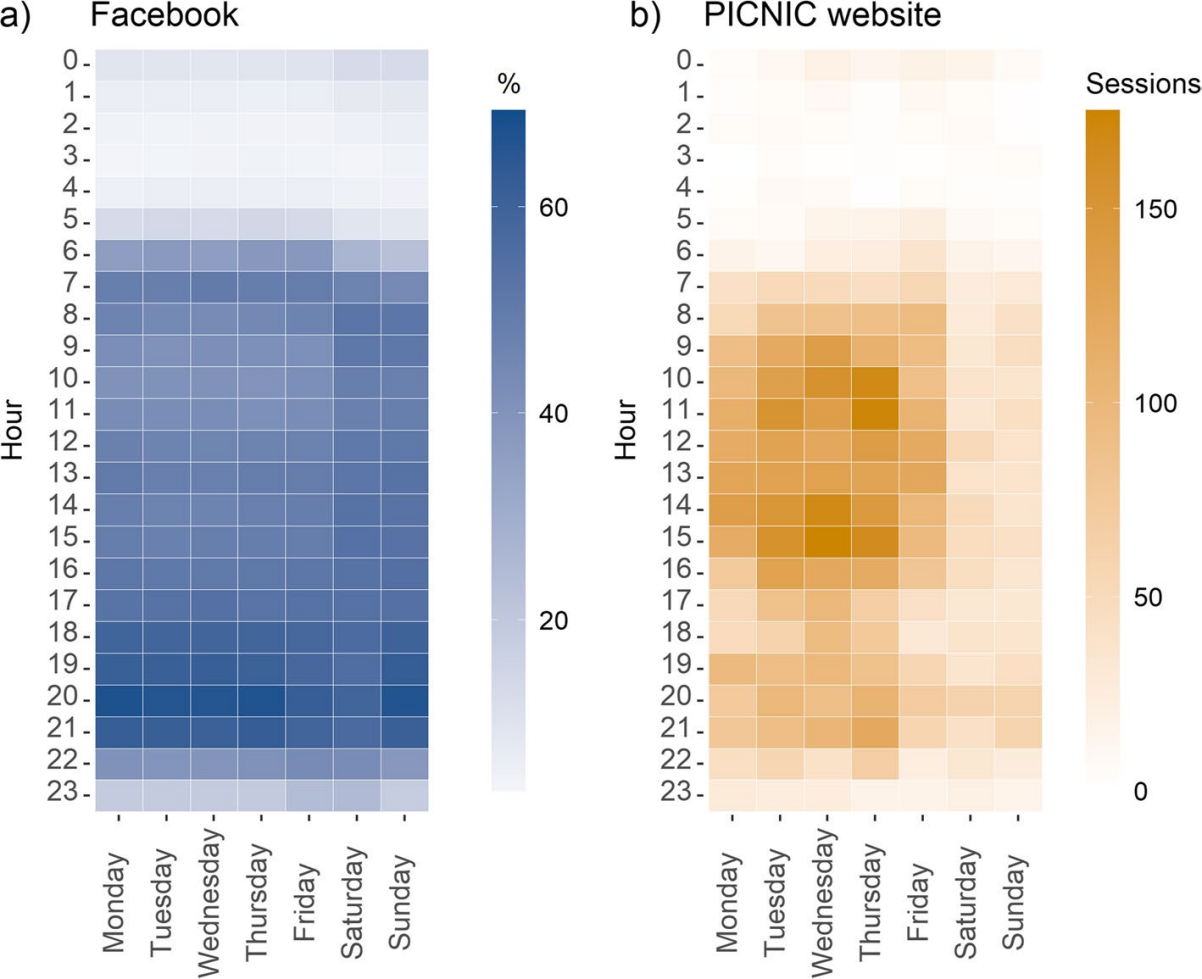
Audience online. Heat maps showing **a**) the percentage of Page fans (who had liked the PICNIC Fb Page) that were online on each specific hour with average data for each weekday and hour, and **b**) PICNIC website sessions by time of day and day of the week (total number of sessions/hour over the 1.5-year period)

In order to increase reach to the intended PICNIC audience digitally, we sought to understand if and when they were online. On average 94.9% of people who had liked the PICNIC Fb Page were online on Fb at some point each day. This number was steady throughout the analysed period (range 92.9–96.9%) with no noticeable differences between months. This reconfirms the high daily use of the Fb platform amongst parents. Furthermore, we extracted data on the number of Page fans who were online on Fb by each hour of each day. No difference in patterns was observed between months; therefore, the data was combined into a single heat map representing the entire period (Fig. 6a). Fb Page fans appeared to visit Fb regularly throughout the day and the week, with most activity observed during evening hours (7-10 pm). Figure 6b shows the total number of website visits by hour and day with data from the entire evaluation period. Contrary to Fb activity it appeared most website visits occurred during office hours (8 am to 5 pm), and less on weekends.

### Demographics of ‘reached’ audience

**Fig. 7:**
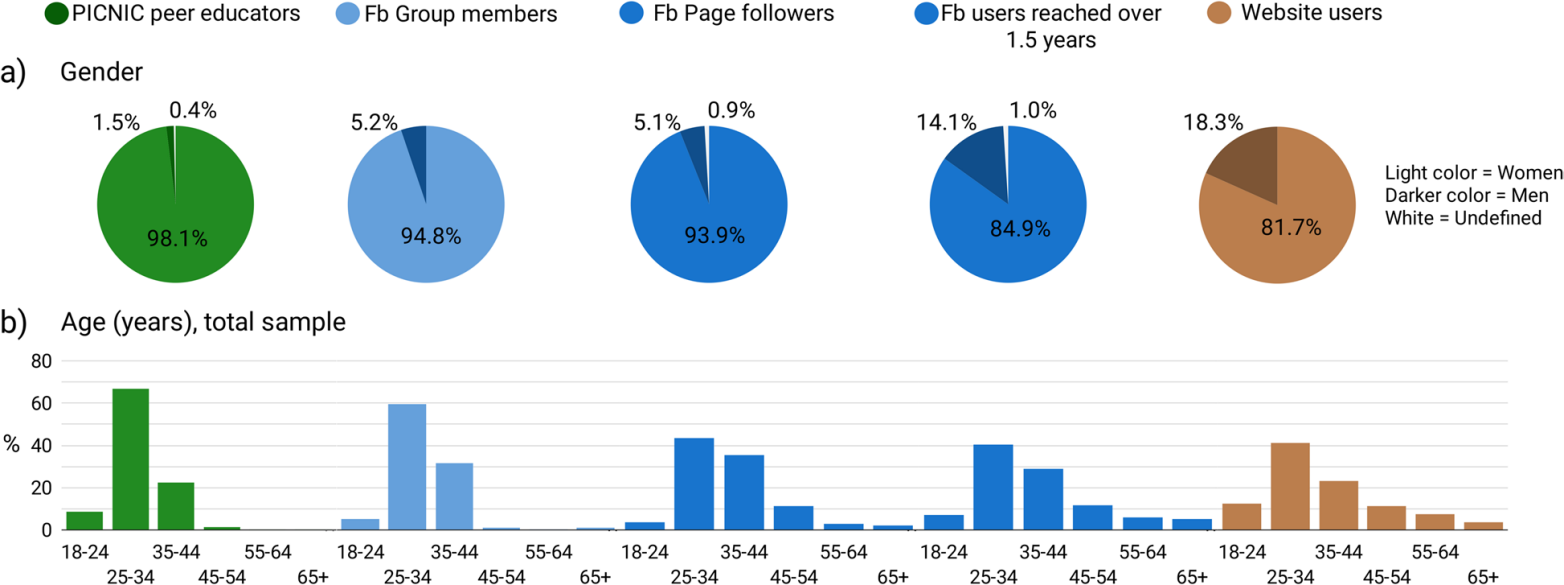
Audience demographics. Bar and pie chart showing age and gender distribution of peer educators versus the audiences reached through the different online channels. Gender and age data was available for 100 and 78% of peer educators, respectively, all Fb group members and Fb Page followers, and almost all (> 98%) of Fb users reached by the intervention through Fb Page posts. Google Analytics only collects demographics data on a subset of website users (e.g., when DoubleClick cookies are present, or users are logged in to Google), and during the 1.5-year period gender and age information were available for about 37% of PICNIC website users. Fb, Facebook

Figure 7 shows (a) age and (b) gender distribution comparisons between the audience reached through the different channels. The PICNIC online community were predominantly females across all channels. About 1.5% of enrolled peer educators, and approximately one in 20 Fb group members and Fb Page followers, were men. However, the proportion of men amongst all those who were reached on Fb by the PICNIC posts during the observation period were substantially higher (14%), and approximately every 5th user visiting the website from Australia appeared to be male. At the time of enrolment in the PICNIC program, 90% of peer educators were aged between 25 and 44 years. As expected, these were also the most common age groups for the online audiences reached, but Fb Page posts and the website appeared to reach people within a wider age span, with slightly higher age-group representation compared to ages of enrolled peer educators.

### Engagement within the Facebook closed group

During 1 year (May-20 to end of Apr-21) the closed Fb group membership grew from 198 to 307, and a total of 235 posts (4–5 each week) were posted in the group. The majority of the content was posted by the group administrators/moderators (shared post from PICNIC Fb Page, posts welcoming new parents, or other program related posts), and 12% of posts were by parents in the group (sharing tips or experiences, or asking questions on feeding). The number of daily active members (defined as members who viewed, posted, commented or liked/reacted to group content) ranged from 0 to 207 members/day, which corresponds to 0–73.5% (median 5.2%) of the total group members per day. In 1 year, a total of 227 comments and 742 reactions to posts were recorded in the group; however, this also includes those from administrators/moderators, such as answering a member’s post or question.

### Facebook page posts engagement

**Fig. 8:**
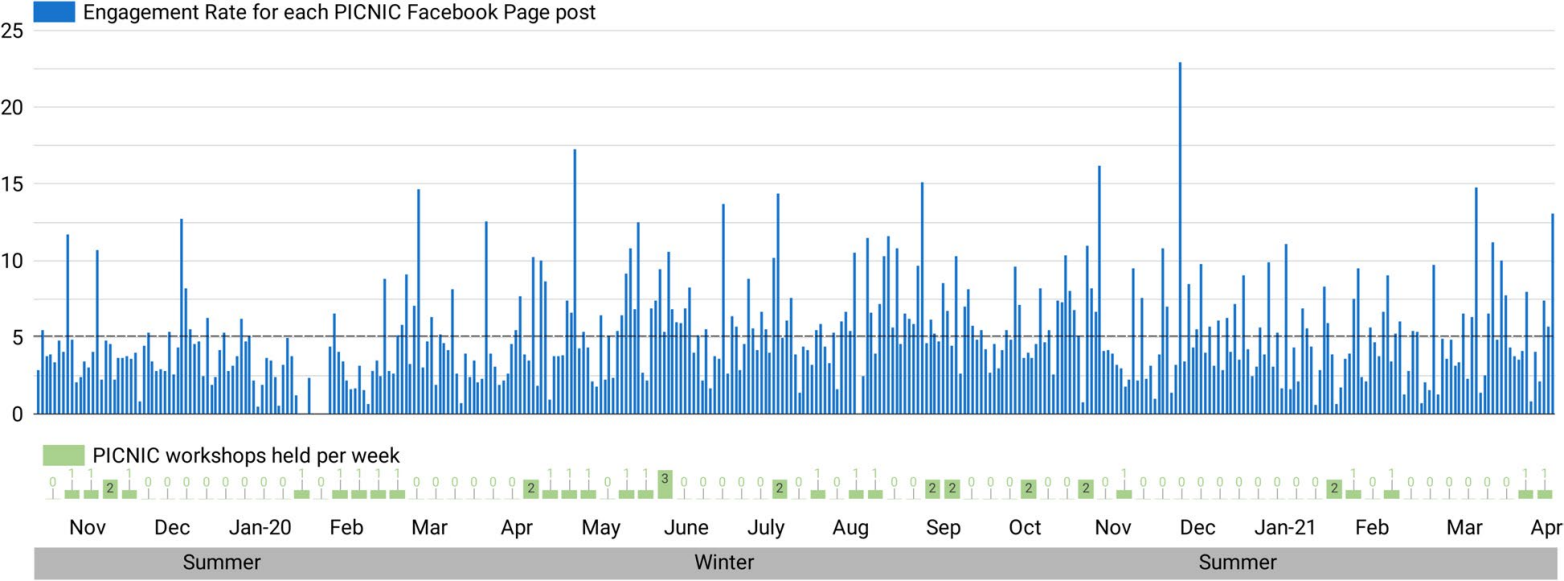
Per-post engagement relative to post reach. Engagement rate, calculated as Lifetime Engaged Users (Unique Users) / Lifetime Post Total reach (Unique Users)*100, is shown for each of the 359 posts plotted in chronological order over the 1.5-year period evaluated. Average engagement rate (5.1% of reached users) is indicated with a reference line

While the closed Fb group existed primarily to facilitate communication between peer educators in the program, the purpose of the open Fb page was to share information around child feeding to be disseminated to parents and their peers. The Engagement Rate for each of the 359 Page posts (299 photos, 23 videos, 22 link posts, 15 text posts) posted by the PICNIC research team over the 1.5 years, is shown in chronological order in Fig. 8. This metric estimates how many of those who saw the post (i.e., Total Post Reach) also chose to engage with it (commenting/liking/sharing or clicking on the post). The Engagement Rate varied greatly from post to post, averaging 5.1%. This means, if a single post is shown to 400 people, approximately 20 of them will engage with it. Engagement rate for the five boosted posts (including two videos) varied between 0.7 and 9.9%. Furthermore, by looking into metrics for the different types of engagements that were triggered by the posts, on average 26 users clicked somewhere in the post (‘consumers’) and 13 users per post created a ‘story’ about it. Consumptions included: clicks to view photos (24% of all post clicks), link clicks (9%), video plays (5%) and other types of post clicks (63%) such as expanding to read the post caption or comments, clicking on someone’s username, or the ‘like count’ to see post reactions. Stories included: liking/reacting to the post (72% of all stories created), sharing (17%), and commenting (11%) on the post. Ninety-four percent of all posts were ‘liked’ at least once, and 41% (146 posts) received 10 or more likes/positive reactions.

### Arriving at the website – user acquisition

**Fig. 9:**
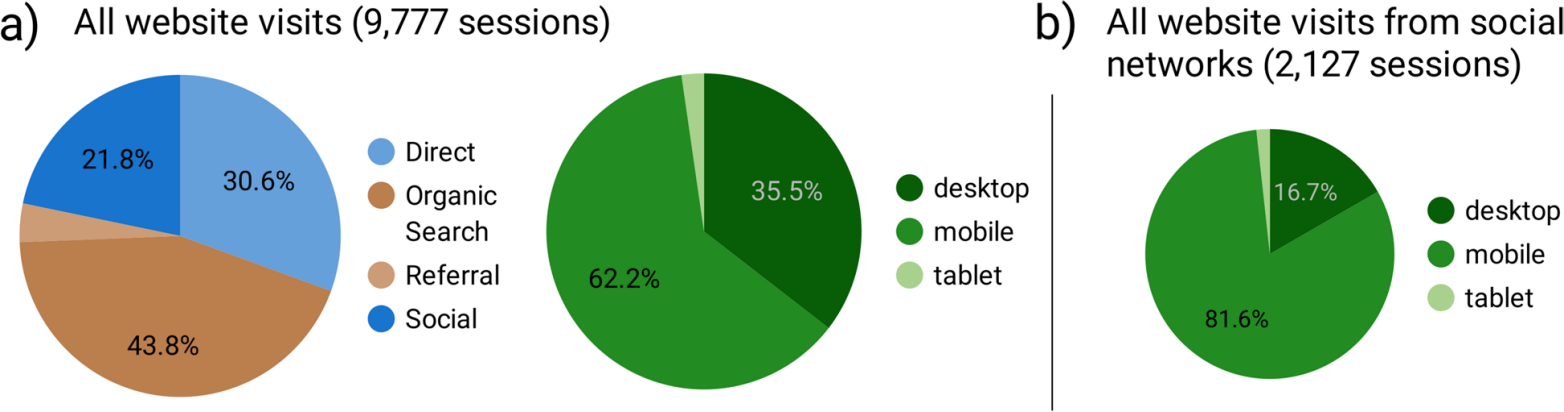
Acquisition channel and device used to visit the PICNIC website. The pie charts are based on visits i.e., sessions, not individual users, as people may arrive at the website in different ways from visit to visit. Direct: users arriving through a source not recognisable to Google Analytics (such as typing in the URL directly, using browser bookmarks, clicking a non-traceable link in a document or e-mail, and other). Organic search: users arriving through a search engine. Referral: users referred to the website through a traceable link clicked on another website. Social: users arriving from a social network platform, such as Facebook or Instagram

During 1.5 years, the PICNIC website had a total of 9777 visits (sessions) from users within Australia. About half of these (52.5%) were recorded as first-time sessions. Based on all tracked sessions, the most common way to enter the website was by organic search (e.g., Google or other search engines), which acquired almost half of the visits (Fig. 9a). Every fourth visit came from social media platforms, with Fb accounting for the majority (92.9%) of these. Users arriving from social media more often used mobile phone devices (81.6% of sessions, Fig. 9b). Finally, the rest of the visits were either traceable referrals (i.e., users clicking through on a link at another website) or classified as ‘Direct’ traffic. The latter includes those arriving by typing the website URL directly into their browser or by using browser bookmarks, but also when sources are unrecognisable to GA (for instance, a link in an Outlook e-mail, PDF or other document).

### Most viewed PICNIC website pages

**Fig. 10:**
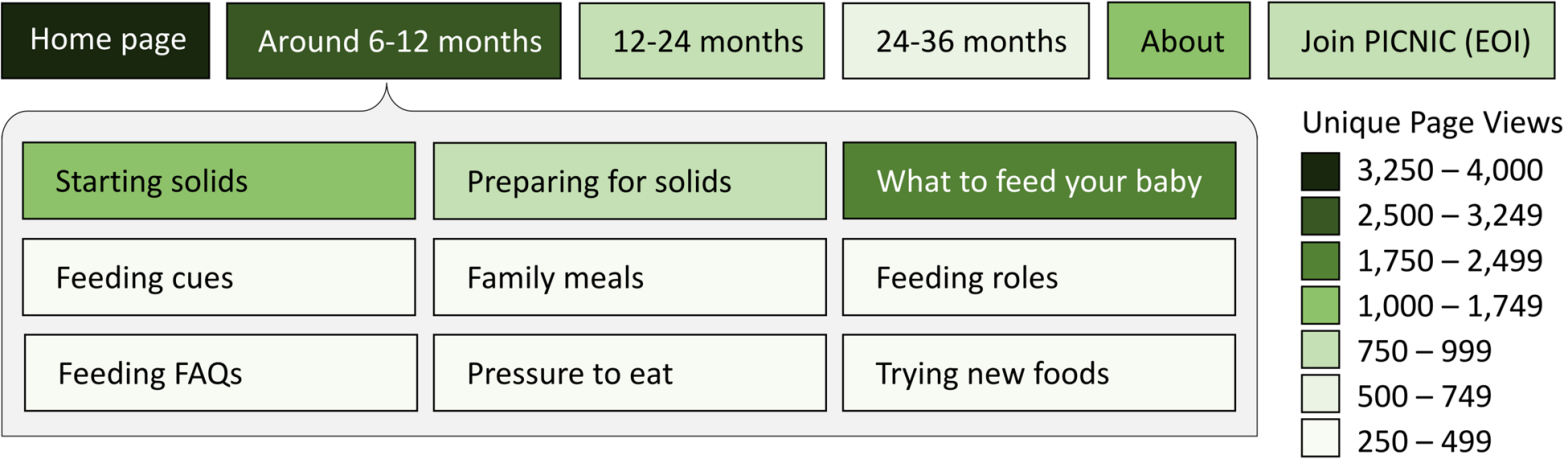
Most popular pages viewed by visitors to the PICNIC program website (picnicproject.com.au). Heat map based on engagement metrics obtained in Google Analytics for all sessions by users visiting from Australia during the 1.5-year period Nov-2019 to Apr-2021. The subpages within the age category 6–12 months are shown since this was the most viewed part of the website. Unique page views: the number of sessions during which the specified page was viewed at least once, based on URLs. EOI, Expression of Interest

The primary aim of the PICNIC website was to provide a repository of evidence-based child feeding information to parents and other caregivers. As expected, the most common landing page was the home page (35.6% of all sessions), which consequently had the highest amount of unique page views (Fig. 10). Among the three feeding information categories, “6–12 months” was clearly of most interest to visitors; its main page had more than three times as many unique page views as “12–24 months” and more than five times as many as “24–36 months”. Popular subjects within this category were “What to feed your baby”, “Starting solids” and “Preparing for solids”, as indicated by relatively high number of unique page views. Over the 1.5-year period, the search function on the website was used a total 137 times by users in Australia. Most search terms were about foods (such as specific food items, food amounts, vegetarian, or milk/dairy terms). Searches also included terms related to feeding practice or eating behaviour information (e.g., “feeding”, “role”, “mess”, “stress”), as well as recipes, baby led weaning, screen time, choking/gagging and allergies/allergens.

### Expression of interest to join the PICNIC program

The second aim of the website was to aid in the recruitment of new parents to the program. Continuous recruitment was performed through Child and Family Health services, parent/caregiver specific programs and social media [15]. Of the total 9777 website sessions observed (5187 users), 9.8% included a visit to the page “Join PICNIC” which provided the EOI form, and in 2.8% of the sessions (275 unique users) the EOI form was also submitted (considered ‘conversions’). This number agreed well to the number of EOI forms received by the Chief Investigator (R.B.): *n* = 281. Segment analysis in GA (Table 1) clearly showed a difference in engagement between those sessions in which users did not visit the EOI page, compared to sessions where the EOI page was viewed and/or the form submitted (i.e., ‘converters’). The latter had lower bounce rate, higher average session duration, and more pages viewed per session, suggesting higher engagement in the website. Also, these ‘converters’ were more often women, acquired from social media, and primarily used mobile devices. In addition, the Landing Page report showed that sessions leading to conversions more often landed directly on the EOI page rather than the home page. This suggests recruitment through social media and the strategy of directing potentially interested parents to the EOI page using links in Fb posts was successful.

**Table 1:** Website metrics comparing sessions with and without indication of conversion to join the PICNIC program

	Sessions – no EOI pageview^a^	Sessions – EOI page viewed^b^	Sessions – EOI submitted^c^
Total no. of sessions (% of all 9777)	8821 (90.2%)	956 (9.8%)	278 (2.8%)
Users (% of all 5187)^d^	4611 (88.9%)	796 (15.3%)	275 (5.3%)
**Engagement metrics**^e^			
Session Bounce Rate	44.9%	27.6%	0%
Avg. Session Duration	3 min 32 s	4 min 59 s	4 min 37 s
Pages/Session	2.79	4.06	3.78
**Gender**^f^			
Women	79.2% (2379)	85.5% (389)	89.9% (125)
Men	20.8% (624)	14.5% (66)	10.1% (14)
**Acquisition**^g^			
Organic search	45.7% (4032)	26.4% (252)	13.0% (36)
Direct	32.2% (2841)	15.4% (147)	9.7% (27)
Social	18.0% (1586)	56.6% (541)	76.3% (212)
Referral	4.1% (362)	1.7% (16)	1.1% (3)
**User device**			
Mobile	61.3% (5409)	70.7% (676)	92.5% (257)
Desktop	36.5% (3223)	25.6% (245)	6.1% (17)
Tablet	2.1% (189)	3.7% (35)	1.4% (4)
**Top Landing pages**			
1	Home page (34.9%)	“Join PICNIC” (43.3%)	“Join PICNIC” (54.3%)
2	“About” (15.9%)	Home page (20.6%)	Home page (10.1%)
3	“What to feed your baby” (10.0%)	“About” (10.7%)	“About” (9.0%)

*EOI* Expression of Interest

^a^All sessions which did not include a view of the EOI (“Join PICNIC”) page

^b^Sessions were the EOI page was viewed

^c^Sessions were the EOI page was viewed and the form submitted (as defined by a page view of the thank-you page)

^d^Count of unique users behind the sessions in that category

^e^Bounce rate is the percentage of single-page sessions. Average session duration is calculated for non-bounce sessions, i.e., includes only sessions with more than one page viewed. Pages/Session is the average number of pages viewed, including single-page sessions

^f^Gender information is only available in Google Analytics for a subset of users (see Methods and Additional file 1)

^g^Acquisition channel from where the visit arrived to the website from. Gender, acquisition and device data is provided as percentage (and count) of total sessions in that specific segment

## Discussion

This study identified substantial advantages of actively using both closed and open forums for online social networking in a peer education project to reach and engage parents with evidence-based child information. Web and social media analytics from PICNIC [15] showed steady program growth in the 18 months of recruitment since November 2019. Participant numbers in this period grew from 102 to 261 peer educators while the Fb Page audience increased threefold, totalling 1615 PICNIC followers by the end of April 2021 in this ongoing program. At the time of writing (Jan-22) the Fb PICNIC audience has further increased to 353 group members and 1833 Page followers. This study therefore provides benchmark estimates for future comparison.

### Closed peer group engagement

Few studies have incorporated a social media component in a child health intervention targeting parents of infants [26–29], or preschool-aged children [30–32]. These studies typically used discussion groups on social media for information sharing between study participants, and report varying levels of parental engagement and satisfaction. Barriers reported include modest engagement in Fb groups by parents of preschoolers [44], low agreement that the Fb group component was useful [32] and waning interest in Fb group over time [28]. Fb groups were only used to complement a more complex intervention in these studies, which may have influenced participants’ engagement in the group. In an obesity prevention program targeting low-income mothers with overweight or obesity [27], the research team actively used Fb groups to encourage interaction with a video-based curriculum and online activities, with high parent engagement (30 participant posts or comments/group/week) and satisfaction reported. PICNIC receives a similarly high number of recorded comments and reactions each week (average 19 comments or reactions, including PICNIC team comments), with 12% of posts (average of one every 2 weeks) by parents. Intervention content was simultaneously posted in public spaces (Fb Page and Instagram), so parents also had the option of engaging their instead.

### Using public social media page to reach parents’ peers

Public social media channels have fewer privacy constraints, so more analytic data points are accessible to evaluate the reach of campaigns, including those aimed at new parents [29]. The public PICNIC Fb Page is used for dissemination of messages and for recruiting new parents to the program, and has six times more followers compared to the number of enrolled PICNIC parents. Fb Page content (primarily organic posts) often reached more than a thousand unique Fb users each week, a substantial proportion of the primary PICNIC target population of approximately 8800 parents with a child under 2 years in the region [39]. The Breastfeed4Ghana [29] and ‘Make Healthy Normal’ campaigns [45] reported that paying for posts helped boost reach and follower acquisition. However, they also conclude that content still needs to be engaging to the audience and that paying for posts not necessarily predict higher engagement [29, 45]. So far, PICNIC has used primarily organic content, and although boosted posts (*n* = 5) received extra reach they did not consistently outperform other posts. Future decisions about which PICNIC posts to boost or pay for will be informed by ongoing comparison of reach and engagement of paid versus organic content and the intended audience for specific posts.

Despite the overall high reach of PICNIC Fb posts, each Page posts typically only reached a quarter of all Page followers and were only seen once. Recycling of posts (with some rewording to avoid being penalised by the Fb algorithm) and posting in the early evening to capitalise on evening Fb viewing patterns are likely to increase exposure, whilst substantially reducing resource development time. Regularly posted content may function as a good reminder to parents, keeping them on track with child feeding. It will increase likelihood that parents are exposed to particular messages at a suitable time in their child’s development, which is a vital consideration in child feeding and consistent with emerging ‘just in time’ teaching and learning methodology [46].

Social media reach is heavily influenced by post engagement [34]. Although some PICNIC Page posts spread considerably on Fb through the ‘peer-to-peer’ viral reach, on average 5.1% of those who saw PICNIC Page posts chose to actively engage in it (clicking/liking/commenting/sharing), and the level of engagement varied considerably from post to post. Another potential future strategy to increase reach is encouraging PICNIC parents to engage with PICNIC Fb posts actively but discriminately, which will serve a dual purpose of increasing reach and increasing the sensitivity of analytic data.

Engagement in eHealth behaviour interventions is defined and captured differently depending on the purpose and context of the research [47], and the audience and platform used [33, 48, 49]. Engagement is considered necessary for achieving behaviour change in eHealth interventions [47], although it may not have to be ‘active’ engagement alone; lurkers (‘silent’ users who view without clicking) can still be active consumers of content [50]. Consistent with the findings by Ellison et al. (2020), it is likely that PICNIC parents consider how active engagement will be perceived by their network or how it will inform the Fb algorithm that influence their News Feed [50]. A PICNIC social media content analysis would improve understanding about which types of posts and content achieve higher reach and engagement. This content analysis could compare analytics data with qualitative data to better understand what type of content is appreciated and useful to parents. The processes for conducting content analysis and findings regarding child feeding-related social media would be generalizable and applicable internationally.

### PICNIC website

The primary function of the PICNIC website is as a repository for evidence-based child feeding information. Page views and search terms indicate visitors were more interested in what to feed and how to prepare solids, rather than responsive feeding practices. This illustrates that parents of infants 6–12 months may not yet be concerned about fussy eating or other feeding issues. However, PICNIC aims to embed responsive feeding knowledge to these parents in the early stage (also at the education workshop) to prepare parents in advance about what to expect. This anticipatory guidance approach is consistent with a similar infant health intervention [51], and is important since parental behaviour and feeding practices influence child eating behaviours [7, 8]. For instance, Ek et al. [52] showed that parental pressure to eat was strongly associated with children’s food avoidance at around 5 years of age. It is possible that PICNIC social media posts may be useful to continue informing and reminding parents about these topics, but whether the PICNIC intervention is enough to positively influence parents feeding practices and child eating outcomes is yet to be determined. Further strategies may be explored to encourage parents to return to the website for additional information when their child is getting older. To better understand users’ behaviour on the website complementary tools could be used, for example tracking clicks and scrolling on pages using Google Tag Manager [53] or Hotjar [54].

The relatively high proportion of user acquisitions from organic search reported in this study (43.8%) is consistent with that reported for other health or parenting information websites. For instance, 51.9% of visits to ‘No Money No Time’, a healthy eating website targeting young people in Australia [55], and as much as 90% of visits to babysleep.com, an international baby sleep information website [56], were acquired through search engines. Considering parents often seek parenting information on the Internet [10, 13], this highlights the importance of search engine optimization (SEO) [57] to make sure users find the website when searching for a relevant topic online.

The PICNIC web analytics results suggest that recruiting through Fb worked well, as 76% of those who filled in the EOI form on the website were referred from social media, often by clicking a Fb post link to land directly on the ‘Join PICNIC’ page. As we report elsewhere [16], this recruitment strategy has evolved over time and became successful because PICNIC parents were willing to on-share their positive experience and refer their peers to the program. This finding is consistent to previous literature by Collins and colleagues [58], who compared recruitment strategies for young women in three nutrition studies and concluded that advertising the study through social media facilitates recruitment.

The upturn in online activity in PICNIC during Covid-19 is likely to have resulted from a combination of factors. Education workshops were moved from face-to-face to online, which resulted in higher attendance rate and increased partner participation [16]. The physical distance restrictions and lockdown during the pandemic may have also contributed. Increased use of social media as a medium for interacting with peers, family, and social groups, and as a source of health information have been reported during the pandemic [59]. Interviewed women who became mothers in Australia during the pandemic described a strong feeling of isolation, and reported seeking and providing social peer support through social media, often Fb [60]. These findings are likely to be generalizable to women worldwide in countries where social media contributes to inter-personal connections.

The ‘open’ online PICNIC resources (public Fb Page and website) reached a higher proportion of men, and a wider age span compared to enrolled participants who were primarily mothers. Social media has been identified as a source of parental information to mothers [14], however less is known about how fathers seek parental advice online, and men are often underrepresented in child health interventions [61]. In PICNIC, fathers sometimes attended the online workshop together with their partner but did not formally enrol in the program. Inclusion of fathers has positively influenced retention and engagement of mothers [62] and maternal and infant health indicators [63] in parenting programs. Thus, strategies should be explored to involve more fathers in PICNIC, as well as grandparents or other family members who may influence child nutrition practices [64, 65].

### Strengths and limitations

Strengths of this study includes the relatively long evaluation period (1.5 years), which covered the most intensive recruitment period in PICNIC so far, and also enabled consideration of contextual factors such as seasonal changes and pre−/during the Covid-19 pandemic. The data was objectively measured, which limited the risk of recall bias and enabled us to understand the online dissemination of intervention content over time and across platforms. We were able to evaluate both social media engagement and website usage for exactly the same time period, enabling better understanding of these resources in the program. Since Fb has been the most used platform for parents to seek child feeding information, both in PICNIC and in general [14], we focused this evaluation on Fb. The use of Instagram and potentially other networking platforms will be monitored and explored to ascertain social media preferences of the target audience. Access to data from the open PICNIC website and social media Page provided greater opportunities for analytics analysis because considerably more data is provided by Fb for public pages than for groups, which are restricted for privacy. The platforms and analytics tools used in the current study are freely available worldwide. However, there is limited evidence available to inform the use of social media and its associated analytics tools within child feeding and nutrition interventions currently. The findings from this study regarding use of social media and web analytics will be applicable to any country in which researchers are measuring program reach and engagement of interventions with a similar social media component.

This study had some limitations. Although we could ascertain that 97% of Page followers were from Australia, it was not possible to filter all Fb data based on location of users. Since only aggregated data on group level was used, we could not identify or filter out specific individuals. It would be interesting to investigate the association between participants’ online engagement with dietary and feeding behaviour outcomes of the study, but that is beyond the scope of this study and would require further ethical considerations. There were also some limitations that stemmed from how website [42] and social media data is measured or estimated, which have been outlined in detail in Additional file 1. While this study provided insights into how social media algorithms impact on content reach, knowing how to ‘feed’ these algorithms is challenging because they are not transparent and are constantly updated [34, 66]. Also, parents communicating and sharing of information through e.g., Messenger, emails or face-to-face was common in PICNIC [16], but was not captured by analytics data.

In GA, there is substantially more data available than what we report in this study. Since GA is developed for marketing and sales purposes, the reported metrics need be interpreted with caution when applied to a research evaluation project [42]. However, this study has provided an estimate of the reach and use of the website, and has informed improvements for further assessment, such as filtering website traffic based on specific IP addresses, additional settings and migrating from GA Universal to GA 4 [67].

Finally, analytics can help describe users’ behaviours on a platform and their interactions with the content but cannot be used to understand the ‘why’. Therefore, the results from this study will be used as a ‘piece in the puzzle’, considered together with qualitative findings from the PAR process [16] and interpretation of the PICNIC intervention outcomes to better understand the mechanisms of impact [35, 36].

### Recommendations

To succeed online in digital marketing, a data-driven approach is necessary, but minimal guidance exists about how to use web and social media analytics to evaluate and improve a health intervention. Strategies, choice of measurements, and correct interpretation of data depend on the type of intervention and specific aims, platforms used and expected engagement by participants. Conducting this study provided valuable learnings regarding analytics for research purposes, which we have summarised in Table 2. In addition to PICNIC specific recommendations, this table provides general considerations for using web and social media analytics that is applicable to any type of online health intervention that includes a planned social media component. We have also been guided by useful recommendations of others about online recruitment [58], the methodological and ethical consideration of using a website and/or social networks for health promotion or behaviour change interventions [22, 25, 37, 55, 68, 69], and the application of social marketing principles [35, 70]. Despite challenges, web and social media analytics can provide useful insights into intervention program content and strategy improvements. To aid other researchers who wish to explore web or social media analytics, we chose to visualise most of our results, and provide an extensive ‘data dictionary’ [see Additional file 1] of common terms and dimensions, to optimise data interpretation.

**Table 2:** Considerations for using web and social media analytics when planning and conducting online health interventions

Considerations	Recommendations for PICNIC
**Get to know the data and tools** – analytics tools are often developed for business/marketers; be mindful with interpretation of metrics and dimensions when applied for other purposes	Consider migrating to GA4, and possibly explore also other types of tools such as a tag manager system and behaviour analytics tools (e.g., Hotjar).
**Define your questions for useful insights** – specific research questions are required; balance with exploratory work to identify suitable tools, metrics/dimensions and preliminary insights – use the data to evaluate the program, understand best practice, and to answer specific questions; then implement gained insights to improve your program	Continuously monitor growth of PICNIC using analytics data (using customized dashboards). Explore specific questions and compare results to current study.
**Consider time, resources and required expertise** – analytics are time consuming and may require external expertise, allocated resources and cross-disciplinary collaboration	Expand and value collaborations and networking, and optimise use of skillset within existing extended team.
**Know your audience and involve them** – use co-design to involve users in the development and design – combine data sources (such as analytics and qualitative feedback) to understand and align to users’ needs, preferences and behaviours online	Continue participatory action research approach, including qualitative study to further evaluate users experience and perception of PICNIC social media strategies.
**Work with social media and content strategies** – understand how the platform and your intervention is used by the audience, and monitor trends in social media algorithms that may impact content reach – set-up or improve content-, posting- and marketing strategies; for instance, identify ways of increasing post engagement, and consider strategic use of targeted paid posts in combination with predominantly organic content	Conduct a content analysis and combine with qualitative data to understand what type of content is more engaging and/or useful to parents. Include more targeted paid/boosted posts to complement organic content, and evaluate their performance. Since a high portion of website visitors arrive through organic search, also address SEO.
**Keep updated in a rapidly evolving field** – changes can provide new opportunities (e.g., developed platforms or useful tools), but also challenges (e.g., tools removed, updated terms or use, changes in how metrics are measured/estimated, audience migrating to a different platform)	Monitor parents transitioning to other social media platform (e.g. Instagram, TikTok) by monitoring key metrics (reach, engagement) as well as feedback directly from parents. Regularly explore new tools and updates.

The table provides general considerations for using social media, and web and/or social media analytics, in the planning and conducting of online health interventions, as well as related recommendations specific to the PICNIC program

*GA4* Google Analytics 4, *PICNIC* Parents in Child Nutrition Informing Community, *SEO* search engine optimization

## Conclusion

This study has shown it is possible to reach and engage parents in evidence-based nutrition and feeding information by meeting them ‘where they are’ on social media. It has also demonstrated the potential of utilising both web and social media analytics to gain useful insights into the growth, reach and user engagement in the program online. To the best of our knowledge, this is the first study where both website and Fb analytics over a 1.5-year period have been explored to gain insights into a child feeding intervention delivered to parents and their peers through social media. The insights gained in this study will be used for PICNIC program development and refinement of online strategies to further improve the support to new parents.

## Supplementary Information

**Additional file 1.** Data dictionary: analytics terms and measures explained. Data dictionary table with relevant Facebook and Google Analytics terms and measures defined and explained, and commented upon in relation to their use in the study and/or general considerations.

**Additional file 2.** Supplementary Methods: Analytics processes for web and social media components of a population-level child health and nutrition intervention. Supplementary Methods providing further details of the analytics processes performed in the study.

## Data Availability

The datasets used and/or analysed during the current study are not publicly available due to ethical constraints but are available from the corresponding author on reasonable request.

## Abbreviations

Fb: Facebook
GA: Google Analytics
NSW: New South Wales
PAR: Participatory Action Research
PICNIC: Parent in Child Nutrition Informing Community
