# Supplementary material for: Online reach and engagement of a child nutrition peer-education program (PICNIC): insights from social media and web analytics
Source: BMC Public Health. 2022 Apr 26;22:836. doi: 10.1186/s12889-022-13252-3 (PMC9041288; doi:10.1186/s12889-022-13252-3)
Supplement: Supplementary file 1 — Additional file 1. Data dictionary: analytics terms and measures explained. Data dictionary table with relevant Facebook and Google Analytics terms and measures defined and explained, and commented upon in relation to their use in the study and/or general considerations. [file 12889_2022_13252_MOESM1_ESM.pdf]

Online reach and engagement of a child nutrition peer-education program (PICNIC): insights from social media and web analytics.

Additional file 1

Data dictionary: analytics terms and measures explained.

**Term/dimension/metric***	**Explanation**	**Notes on the use in PICNIC and general considerations**
**FACEBOOK (Fb)**		
Group terms:		
Fb Group	A closed place within Fb where members of the group can communicate around a subject with each other and the group admins or moderators. Groups can be private or public. Anyone with a Fb profile can create a group.	PICNIC is a private group, which means only current members can see what is posted, commented and shared within the group.
Facebook Group Insights	Analytics data available for groups with >50 members, providing information (metrics) about the group, such as number of members, activity and engagement within the group, and users demographics (>250 members).	Group Insights can be viewed by admins directly on Fb (28 last days shown as default) and exported. Data up to 1 year back can be exported, except some metrics which is only provided for the last 28-day period from the day of data exporting (‘top engaging posts’ and ‘top contributors’).
Group member	People who have requested to join the Fb group and had their request approved by a group admin. Members may see content from the group in their news feed.	Group Insight provide daily metrics on total number of members, as well as pending, approved and declined member requests.
Active member	Fb group member who viewed, posted, commented on or reacted to group content (metrics per day)	Daily metrics. Used in the present study as an indicator of the overall engagement of members in the group.
Page terms:		
Fb Page	A space on Fb where a business, brand, public figure, celebrity, organisation or non-profits can communicate their stories and connect with their audience. Anyone who has a Fb profile can create or help manage a Page.	The evaluated Fb Page in the present study (PICNIC Mid North Coast) is a public “Business” page created to provide a resource of posts with feeding information and support that can be easily shared from parents to their peers. The Page was also created to increase awareness of the program, and to recruit more parents to the study.
Facebook Business Suite	A free tool for administrators of Fb Business Pages, where their accounts across both Fb and Instagram can be connected and managed together. Used for activity overview, some insights, creating and scheduling posts and stories, creating advertisements, and more.	So far, Business Suite has had limited analytics insights available compared to Page Insights or the previously available tool Facebook Analytics (removed by Fb on July 1st 2021). Hence, Business Suite was not specifically used in the present study.
Facebook Page Insights	A platform in Fb with analytics data available to admins of Business Pages (but not Community Pages) providing aggregated information on the performance and reach of the Page. These include growth (Page	Page Insight can be viewed by admins directly on Fb but can also be exported (up to 2 years back, up to 180 days per file). Key metrics are typically provided as aggregated daily, weekly and last 28-days metrics, with data on page- or post-level, and video data. A few page-level metrics are “lifetime” metrics, such as the total number of

	followers and fans), reach, impressions, engagements, activity, audience demographic, video views and more.	page followers that specific date ("Lifetime Total Follows"), and most post-level metrics are totals i.e. 'lifetime per post'. Separate sheets with stratified data are also available (see a few examples below in this table). The layout can be customised by admins to include the metrics most relevant to that Page. Note: if merging exported datasets, be aware of potential changes in the layout and organisation of dimensions as they may have been introduced by Fb.
Page-level data	Analytics insights metrics provided for the Page in general, with exported data organised by date. Many different types of insight metrics are available for export, such as Page stories, reach, impressions, engagements, reactions, users' demographics and more.	Each row in the exported data file represents a specific date. Note: weekly and 28-day data is still provided for each date; therefore, caution must be taken when summarising these types of metrics.
Post-level data	Analytics insights metrics provided for each specific Page post instead of day, with exported data organised by post.	Data for up to 500 posts and maximum 180-days period can be exported at a time, and up to 2 years back in time.
Video data	Analytics insights metrics provided for each video posted on the Page, with exported data organised by video post.	Data for up to 500 video posts and maximum 180 days period can be exported at a time, and up to 2 years back in time.
Page likes/fans	People who have liked the Page by clicking the "like" button are connected to the Page and called Page fans. Someone who likes a Page will automatically follow it but can actively choose to unfollow while still liking it (a way of showing support without getting the page content in their news feed).	On Jan 6th 2021 Fb announced the New Pages experience, with some changes in design and features from classic Pages; https://about.fb.com/news/2021/01/introducing-the-new-page-experience/ Page likes are no longer used/available in this format, only Page followers. This will consequently affect also the way some metrics is reported.
Page follower	Page followers are those who have chosen to see the Page's updates and posts in their news feed. Usually followers are also Page fans ("like" the Page) but can also choose to follow without clicking the "like" button.	The numbers of Page fans and followers are often very similar. In the present study, we focused on followers since this is arguably the most relevant (have chosen to want to see Page content), and will be kept by Fb in the New Pages experience.
Unlike	A Page fan who chooses to 'unlike' will no longer be associated to that Page as a fan.	"New likes" in relation to the number of "Unlikes" can be used to assess the growth of Page fans during a specified period, but make sure to also consider "Lifetime Total Likes".
Unfollow	A Page follower who chooses to 'unfollow' will no longer see Page content in his/her news feed.	"New follows" in relation to the number of "Unfollows" can be used to assess the growth of Page followers during a specified period, but make sure to also consider "Lifetime Total Likes".
Fb post	Posts include statuses/texts, photos, links, videos and more, and can be original (created and uploaded by Page/group admin or a Fb user) or shared (i.e. re-posting someone else's post).	When a public post is published on Fb it will be shared to the News Feed, but also be visible on that person's profile, or on the Page if posted by a Page admin. Content posted in closed groups cannot be shared publically to members outside that group. Posts on Fb are available until they are deleted.

Fb Stories	Fb stories is another way of sharing content to Fb, and can be in the form of a photo, video (typically 20 sec. of less), Boomerang (animation made of several photos) or live video broadcast. Stories are different than regular posts as they appear above the News Feed (i.e. above the posts), and are not affected by the Fb algorithm in the same way. Stories are visible for the selected audience (Public/Friends) only for 24 hours.	Stories was introduced on the platform by Fb in March 2017. After 24 hours the story can be saved in a personal 'story archive' if that is turned on by the creator of the story. So far, Page Stories has not been used in the PICNIC study.<br><br>Note: Fb stories are different to "Post Stories" which is a metrics term to describe engagement with a post (see below).
Organic content	Non-paid content.	The vast majority of content on the PICNIC Fb Page has not involved payment to Fb to boost post exposure.
Paid content	Content that is paid in some way, such as paid advertisement (Facebook Ads) or boosted posts.	In PICNIC, all posts were originally posted as organic content. However, three were then "boosted" (to try out the function). Boosted posts will appear higher in people's News Feed.
Reach dimensions/metrics:		
Reach	*Page-level:*<br>The number of unique users who had any content from the Page or about the Page enter their screen. This includes posts, check-ins, ads, social information from people who interact with the Page and more.<br>*Post-level:*<br>The number of unique users who had a specific post enter their screen.<br>Reach can be paid or organic (including non-viral and viral), and is an estimated metric.	*Page-level:*<br>The metric "Total Reach" includes organic and paid reach of Page content, while "Total Reach or Page Posts" is the reach of the Page posts only. In the current study, these metrics are very similar since the PICNIC Fb page content were primarily posts. Page-level reach is typically summarised daily, weekly or monthly (last 28 days).<br>*Post-level:*<br>"Lifetime Post Total Reach" is the post-level metrics describing the total reach of each Page post from the time of posting until the date the data was exported from Facebook Insights.
Paid reach	The number of unique users who saw a post, or Page content, through paid distribution (e.g. Facebook Ads advertisements or Boosted posts).	Note, if a person is reached by a post through both organic and paid distribution, they will add to the metrics for both organic and paid reach. Therefore, Paid Reach + Organic Reach does not always equal Total Reach.
Organic reach	The number of unique users who saw a post, or Page content, without any paid distribution like advertisements.	It may be difficult to reach a lot of users through organic reach only, partly because of the Fb Algorithm. Typically, organic Page posts are shown to only a portion of Page followers initially. If users engage with the post, Fb will then show it to more people. In PICNIC, parents are encouraged to 'like' and 'share' posts as a strategy to increase organic reach so that more of their peers can see the posts (part of a peer educators' role in the program).
Non-viral reach	A type of organic reach describing the people reached directly by the posts. These are people associated with the Page, such as Page followers or fans, who saw the post in their news feed or by going to the Fb Page directly.	Reach can be compared with the total number of Page fans/followers to get an estimate of how well the content is disseminated on Fb. For the purpose of the present study, we explored non-viral reach in relation to Page followers, as it gives an indication of how many of the Page followers are reached by the Page posts. On Page-level, non-viral reach percentage was calculated as the average "Daily/Weekly Nonviral reach" /

		"Lifetime Total Follows", and on post-level, "Lifetime post Nonviral Reach" / average no of followers during the period.
Viral reach	A type of organic reach describing the people who saw the post or Page content because their friend likes or follows the Page, shared or engaged with a Page post, etc.	In the present study, the viral reach was used as an estimate of the number of people reached because their friends interacted with PICNIC, i.e., a 'peer-to-peer' reach.
Post impressions	The number of times your Page's posts entered a person's screen. Similar to reach, impressions can also be categorised as organic, viral/non-viral, and paid.	Since a post can be viewed multiple times by the same user, total impressions (total views) is often higher than total reach (number of people reached). For instance, if a post is viewed 3 times by the same user, this will count as 1 reach and 3 impressions.
Engagement dimensions/metrics:		
Post engagement	The number of unique people who interacted/engaged in certain ways with your Page or Page post(s). This includes both creating stories about the posts (commenting on, liking, sharing), and clicking on particular elements of the post ("consuming" the post).	*Page-level:*<br>"Page Engaged Users" are the number of people who engaged with the Page during the period (typically daily, weekly or 28 last days). This includes any click or story created.<br>*Post-level:*<br>"Lifetime Engaged Users" are the number of unique people who engaged in the Page post, for example by commenting on, liking, sharing, or clicking on particular elements of the post.
Post stories	In analytics, Stories is a term used to describe actions (e.g., comments, shares) or reactions (e.g., likes) generated about a post by users who view the post. It does not include other types of clicks such as link clicks, video plays etc.	*Post-level:*<br>"Lifetime Post Stories" is the total number of stories generated about the post (i.e. total count), while "People Talking About This" is the number of unique users that created a story about the post by interacting with it.<br>Stories in this context is different from the "Fb Stories" (see above) which is a different way of posting content on Fb.
Post reaction	When a user expresses their reaction to a post by clicking either "like", "love", "haha", "care", "wow", "sad", or "angry".	A user can react to a post through the 'Like' button on the post (by hovering over, more reaction options will appear). When post Likes are reported alone (such as in exported Page/Post Insights) this will include all positive reactions (like/love/care/haha).
Post comment	User commenting on a post by adding a text/photo which is then visible to others below the post itself.	Users can either add a new comment to a post or continue and 'answer' another person's comment, creating a comment thread.
Post share	If a post is sharable, a user post to others by clicking the "Share" button on the post. The user can choose to share the post to his/her News Feed or Your Story, or for example to a group or Page, or in a personal message to a friend (Messenger).	The privacy settings of the original posts, and the audience to which it was posted to (e.g., Friends/Public), will determine if and how another user can share the post to disseminate it further. In PICNIC, Fb group posts are only available to members within the group, whereas Page posts are public and sharable by the wider audience.
Post clicks/consumptions	Post clicks other than comments, likes or shares. These can be link clicks, video plays, clicks to view photo, clicking to expand post and read caption or comments, clicks on user names and more.	Post "consumptions" are total clicks on posts whereas "consumers" implies unique users that clicked on the post.

Engagement rate	Measured in different ways, defined by Fb page owner, describing the level of engagement in the audience.	In PICNIC, engagement rate was calculated on per-post level as: (Lifetime Engaged Users (Unique Users) / Lifetime Post Total reach (Unique Users))*100. This indicates how many of the people who saw the post also engaged with it (i.e., commenting on, liking, sharing, or clicking on particular elements of the post).
Aggregated datasheets used:		
Lifetime Follows by Country	Daily number of total Page followers sorted by location (number of followers in each Country).	Used in the present study to see the percentage of followers from Australia.
Lifetime Follows by Gender and Age	Aggregated demographic data about the people who follow the Page based on the age and gender information they provide in their user profiles.	Demographics data will be available when the Fb Page reaches at least 100 Page Likes (or Instagram followers) within the top 45 cities.
Daily Reach Demographics	Daily statistics on the total Page Reach by age and gender.	This is the demographics on the people reached by any content from the Fb Page ('Picnic Mid North Coast' in this study).
Daily Liked and Online by Day	The number of people who have liked the Page (Page fans) and were online on Fb some time that day.	Used in the present study to explore the potential of reaching the intended audience on Fb. At the time of this study, this type of metrics was not available for Page followers (only fans); however, no significant difference would be expected since most people both like and follow a Fb Page.
Daily Liked and Online	The number of people who have liked the Page (Page fans) and what times they are online. Exported data show the number of fans online per each hour (columns) and day (rows) and is presented in Pacific time zone (PST/PDT) as default.	Used in the present study to investigate when (time of day and days of the week) people who were interested in the PICNIC Fb Page were online on Fb. Data was converted to Australian Eastern time before summarised in heat maps. This type of insight can help understand when is the highest chance of reaching the intended audience by posting when they are online.
Lifetime Post Stories by action type	The number of stories (e.g. shares, likes, comments) created about the Page post, by story type.	Used in the present study understand the type of engagement/story that were most common amongst those who viewed and engaged in the PICNIC Fb Page posts.
Lifetime Post Consumptions by type	The number of consumptions (clicks) anywhere in the post on News Feed, by type of consumption. (Total count)	Used in the present study to measure a type of "silent engagement", i.e. clicks that users used to engage in the posts.
**WEBSITE**<br>*General terms:*		
Google Analytics (GA)	A widely used web analytics service by Google, which tracks (using JavaScript codes on website pages) and reports website traffic, performance and user insights. In GA, the data is presented in "reports" organised by Audience, Acquisition, Behaviour and Conversions.	Metrics can be viewed on 'hit'-, session- or user level. To be able to get to insights from the data, segments and filters are typically used in the analysis. In the present study, Universal Analytics was used. However, Google is now recommending to migrate to the new and more powerful version of the tool; Google Analytics 4. Note: GA will sometimes apply data sampling (high traffic cases). In the present study, all reports were based on 100% of website sessions.

Segments	A type of filter that can be applied on the data when viewing GA reports. Segments can be both predefined or customised (with possibility to define multiple filtering conditions) depending on the question that is to be addressed. Segments are filtered on users and/or sessions. Once applied, all reports in GA will be filtered and shown for that specified segment. Several segments can be viewed and compared at the same time.	Using segment in GA is a very useful method to be able to compare metrics and reports between subsets of data (e.g., Returning Users, or Mobile Traffic), as a type of stratified analysis or focused analysis. This helps to gain more useful insights of the website traffic data. In the present study, customised segments were created to filter out traffic outside Australia, as well as to segment the sessions depending on what pages were viewed or not (using URLs for the EOI page and the thank-you page after form submission). Segments can also be used when visualising the data in GDS.
Google Data Studio (GDS)	A data visualisation tool to create interactive and sharable dashboards with graphs and charts, launched by Google in 2016. Must be connected to a source of data, which can be a GA account or other tools or data sources such as Google Spreadsheets. The dashboards are constantly updated against the connected data source.	Used in the present study to visualise the data and create the figures. PICNIC website GA account was linked directly to GDS, and specific segments (such as Australia only) were applied for each plot directly in GDS. Fb analytics data and summary data on the peer educators (e.g., gender % and age groups) that were used in plots were linked to GDS using Google Spreadsheets. At the moment, GDS is a free tool within the Google Cloud Platform.
Session	A unique visit to the website by someone. By definition, a session starts when a first hit on the Landing page is sent to GA. The same session continues when the user browses the website's different pages and ends when the user leaves the website or is inactive for more than 30 minutes.	If a user appears to be inactive on the website for more than 30 minutes, that session will terminate and any future activity will count as a new session. Worth noticing is that a new session always starts at Midnight no matter what. This means that is someone is browsing the website from 23.50-00.10, this will count as two separate sessions. This will therefore affect data uncertainty especially if users are often visiting around Midnight.
User	A visitor who has initiated at least one session during the specified date range.	GA determines unique users by assigning each new visitor a unique identifier. Therefore, a session can be tracked only if the user accepts cookies on the website. Identification is typically based on IP addresses. This means a more accurate definition of a user is "unique browser", as a person visiting on a mobile phone and then a desktop will count as two separate users.
New user	First-time user visiting the website.	When a new user visits the website a unique identified is created by Google (stored in the user's browser cache as a small text file; a cookie) that for instance will be used to link that user to any second visits. Note, if the user deletes the cookies between visits, he/she/they will be counted as a new visitor also next time. In the present study, the number of New Users per week was used to observe new activity on the website and growth of the program.
Returning user	A user who has visited the website before and now visits it again on the same browser and device as before.	Note, during a specified time period, a user may visit for the first time but also come back for a second session. That user will therefore add to the "new user" metrics, but then also to the "returning user". This means that the number of new and returning users should not be summed up to get a total.

Country	Country of the user (browser).	Location of the browser that is used to visit the website is determined by the IP address and will thereby provide an estimation of its actual location.
Age/Gender	Age category of the user: 18-24, 25-34, 35-44, 45-54, 55-64 or 65+ years. Gender is classified as either Male or Female.	GA is only able to collect age and gender demographics information of a subset of all users. For instance, the DoubleClick cookie or Device Advertising ID must be present, or the user needs to be logged in to a Google account.
Conversion/Converter	A conversion is a completed activity or action that is achieved by the user (converter), and that is important for the goal of the business/organisation behind the website.	Typically, macro-conversions are purchases/transactions while micro-conversions are activities such as a sign-up or registration. The definition is completely decided upon by the unique business or organisation and the aim of their website. Different strategies can be used to measure and assess conversion rates, such as setting up Goals in GA. In the present study, EOI form submission (thank-you page at end of EOI viewed) was considered a conversion.
Session metrics:		
Bounce rate	The percentage of all sessions where the user only came to one page and did nothing else i.e., “bounced back” or did not browse any other pages than the landing page.	Note, if a user lands on a page and stays there without clicking further to another page, this will count as a bounce. Those type of sessions have a session duration of 0 even though they may have stayed on the page for a while. This is because no more hits were sent to GA. Note, a high bounce rate is not necessarily “bad” – it depends on the purpose of the website and its pages. If pages are somewhat self-contained, a user may get all they want or need without going elsewhere in that session.
Count of sessions	A metric describing user retention. The Count of Sessions report provides the number of sessions recorded as 1 (a user’s first session), 2 (a returning user’s second session),3 (third session) etc.	This metric may be easy to misinterpret; it does not describe the total number of times a user has visited but simply categorises each session based on whether it was recorded as a first-time, second-time visit etc. This mean that a user that has visited the website 3 times during the specified period will add +1 session to all three first Count of Sessions categories in this GA report.
Session duration	The total time a user spent on the website during a single session.	This metrics is calculated using engagement ‘hits’, that is, requests that are sent to GA when a user interacts with pages on the website. This means that time spend on the last page viewed before exiting the website usually is not included in the session duration, hence, the metrics tends to underestimate actual time on the website. Single-page sessions (bounces) do not send a next-page interaction (hit), and are therefore assigned a duration time of 0 sec.
Average session duration	The average time users spend on the website during a session, based on all sessions during a specified time period.	GA calculates this metrics based on all sessions, including single-page sessions (bounces) which have a duration 0 sec by definition. Considering also that time spent on the last page viewed typically is not included in the session duration, the average session duration presented in GA will most likely be lower than the actual average time spend by users on the website.

Pages/Session	The average number of pages viewed during a session.	This includes also single-page sessions (bounces). High average pages/session indicates that users engage with the website by browsing several pages.
Acquisition:		
Traffic Source/Medium	Source is the specific place (e.g. a specific website) where a user arrives from, Medium is the category of that traffic source and describes how users arrived (e.g. organic, email, referral, or "none" for direct traffic).	Available in GA under the Acquisition report.<br>Note: sources/mediums unrecognisable to GA will be classified as "none" and grouped into the "Direct" acquisition channel.
Acquisition Channel	When several different traffic sources have the same type of medium (e.g. organic, direct or social) they are grouped into a channel. Some of the most common channels are: Organic Search, Direct, Social and Referral.	In the present study, the Default Acquisition Channel grouping (system-defined) was used. It is also possible to use custom channels, which are channel groups defined by the user of GA. Secondary dimensions can be used in GA to look further into each channel, see the top sources and compare data across them,
Organic Search	Visitors arriving at the website through searching Google.com or other search engines.	This only includes non-paid traffic.
Direct	Visitors arriving at the website without a traceable referral source. This can be from using saved bookmarks or typing the website URL directly in the address bar, but also by for instance a link in an Outlook e-mail, in Skype, a mobile app, or a PDF or other type of document.	When interpreting traffic sources, be aware that whenever GA is unable to determine from where the traffic came from, this is also reported as "direct" traffic. Hence, in the reported Direct traffic, some types of referrals may also be 'hidden'. For instance, in PICNIC, a parent clicking a website link in a pdf from the research team would result in a session from a 'Direct' acquisition channel. To be able to track such referrals, UTM code tracking can be used.
Referral	Visitors arriving at the website by clicking on a traceable link on another website.	By using Source as a second dimension in the Acquisition report for traffic channels, the specific websites that drive traffic to the website can be viewed.
Social	Visitors arriving at the website from a social network such as Fb or Instagram.	By using Social Network as the secondary dimension, you can for example see which social network drives the most traffic.
Page metrics:		
Landing page	The first page through which the visitor enters the website.	Typically, the most common landing page would be the home page, but it can also differ depending on from where and how the user arrives to the website.
Page views	The total number of pages viewed. Repeated views of a single page are counted.	
Unique Page views	The number of sessions during which the specified page was viewed at least once.	In the present paper, this metrics was used to explore what pages within the website appeared to be most popular, that is, most often viewed.
Entrances	A per-page metrics showing the number of times a visitor entered the website through a particular page (or specified group of pages).	This metrics is found in the Behaviour report (Site Content – All Pages) and is reported for each page or a set of pages. It may help understand where users land and be useful when comparing the roles of the different pages within a website.

Exits	When a user exited the website from a particular page (or specified group of pages).	The % Exit metrics indicates how often users, who view a specific page, leaves (exit) the website from there. It is calculated as (number of exits)/(number of page views).
Avg. Time on Page	The average amount of time users spent viewing a specified page or screen, or set of pages. Only non-bounce and non-exit sessions are taken into account.	Note, GA can only calculate the time spend on a page if the user then browses further to another page within the website. This means that single-page (bounce) sessions and visits when users exit from that page are not taken into account. Hence, in the present study this metrics was not used, as we expect some users to navigate to a page of interest, read and then exit from there.

Admin, administrator; EOI, Expression of Interest; Fb, Facebook; GA, Google Analytics.
* This table includes the most relevant terms, tools and dimensions/metrics used in the present study in 2021. Note that although terms relevant to the current study are covered here, this is only a portion of what exists. More definitions and latest information on terms, analytics insights and tools is available at Facebook Help Center (https://www.facebook.com/help), Google Analytics Help pages (https://support.google.com/analytics/), Meta for Business – Facebook Blueprint (https://www.facebook.com/business/learn) and Google Academy (https://analytics.google.com/analytics/academy/).
