# Supplementary material for: Online reach and engagement of a child nutrition peer-education program (PICNIC): insights from social media and web analytics
Source: BMC Public Health. 2022 Apr 26;22:836. doi: 10.1186/s12889-022-13252-3 (PMC9041288; doi:10.1186/s12889-022-13252-3)
Supplement: Supplementary file 2 — Additional file 2. Supplementary Methods: Analytics processes for web and social media components of a population-level child health and nutrition intervention. Supplementary Methods providing further details of the analytics processes performed in the study. [file 12889_2022_13252_MOESM2_ESM.pdf]

Online reach and engagement of a child nutrition peer-education program (PICNIC): insights from social media and web analytics

Additional file 2

Supplementary Methods: Analytics processes for web and social media components of a population-level child health and nutrition intervention.

Growth and reach analyses

Data from the online sources was summarized per week in order to observe growth and trends over time, and in relation to the ongoing recruitment, educational workshops held, and the covid-19 pandemic. *Growth* was described as the number of enrolled peer educators during the period, the net increase of Fb Page followers/fans, and new visits to the website. *Reach* on Fb was explored using both per-page and per-post reach and impression metrics (further explanations in Additional File 1). In order to understand to what extend PICNIC Fb posts reached (were shown to) people who followed the Page, an overall estimate (%) was calculated based on non-viral reach in relation to the total number of Page followers across the whole period. This was calculated both on page-level (using non-viral reach of Page content per day or week) and post-level (total per post). Viral reach (a type of 'peer-to-peer' organic reach) was assessed to get an indication of the reach of posts to people who were not Page followers but saw PICNIC Page content in their news feed because their Fb friend engaged in or shared the posts, liked or followed the Fb Page etc. Reach of the website over time was examined using weekly number of sessions (website visits) and unique users.

Next, we sought to understand if and when the target audience was available online. First, daily metrics on the number of Page 'fans' (who had liked the Page) that had been online was compared with the total number of Page fans on that day, as an estimate of the proportion of Page fans that went into Fb on a daily basis during the period. Secondly, detailed data on the number of fans online per hour each day was extracted, with every third month selected to represent different times of year in which fans may have different online behaviours and usage patterns. The data was carefully adjusted to the correct time zone (default in Fb Insights is Pacific time), transformed to percentage of the total number of Page fans, averaged and presented in heat maps. Similarly, data on the total number of website sessions on

each specific hour and weekday was extracted from GA and plotted in a heat map to get insights into when most website visits occurred. The website data was summarized for the entire 1.5-year period and not compared across months, because of relatively few data points (sessions) per hour.

Audience demographics

Gender and age data was compared across the channels and audiences reached: enrolled peer educators, Fb Group members, Fb Page followers/fans, Fb users reached by Page posts, and PICNIC website visitors (Figure 1). The same six age categories were used by all channels. Gender and age information from surveys were available for 100% and 78% of the total 261 enrolled peer educators, respectively. Through Fb Insights, data was available for all Fb Group members and Page followers, and almost all (>98%) of the Fb users reached by the intervention through Page posts. GA only collects demographics data on a subset of website users (e.g., when DoubleClick cookies are present, or users are logged in to Google), and during the 1.5-year period gender and age information were available for about 37% of PICNIC website users in Australia.

Facebook engagement analyses

Engagement from the audience on Fb is considered important, especially with organic content, since more engagement generally means more exposure (post reach) on the platform [1]. An Engagement Rate can be calculated in multiple ways, each with a different method of interpretation. Because posts are typically not shown to all followers, a preferred method is to compare engagements with the number of users who actually saw the post (total reach) [2]. In this study we chose to focus on the per-post engagement and calculated engagement rate as: *Lifetime Engaged Users (Unique Users) / Lifetime Post Total reach (Unique Users)*100*; this gives an estimate of how many of the people who actually saw the post also chose to engage with it (i.e., commenting on, liking, sharing, or clicking on particular elements of the post).

Website engagement and conversion analyses

Website user engagement can be assessed in different ways. Bounce rate, user retention, session duration, and pages/session are user behavior metrics commonly explored in digital marketing. However, the type and purpose of the website will determine what analytics are appropriate to use, and caution should be taken when interpreting the data because of the way some of these dimensions are measured. Thus, web analytics should focus on trends and comparisons rather than single numbers. To address the first aim of the PICNIC website—to provide feeding information to parents—we explored

what pages were most popular. Using heat maps, the total number of unique page views were compared between website pages. The website was also used for recruitment by directing interested parents to the EOI form (located on the "Join PICNIC" page), often through a direct link in a social media post. To evaluate this strategy, we first assessed the number of users that submitted the EOI (considered "conversions" in this context). This was done by using the specific URL for its 'thank you' page. Then, key engagement metrics, gender, acquisition, device and top landing pages in sessions with no interaction with the EOI page were compared to sessions where users viewed and/or submitted the EOI form. This data was compiled in GA using customized segments filtered by sessions and the specific URLs. Furthermore, when websites contain a search function it can be useful to assess the search terms used. This can give insights into what type of information users are looking for, and can be used to improve the website structure/navigation (to make sure people find what they are looking for) or the addition of information (for instance, if users tend to search for a type of information that is not yet provided on the website). In PICNIC, search term data was extracted from the 'All pages' *Behaviour* report in GA, using the data for search results pages identified using their specific URLs. Finally, user acquisition and device used was also summarized based on all recorded sessions.

References

1. Cooper P: How the Facebook Algorithm Works in 2021 and How to Make it Work for You. Feb 10, 2021. https://blog.hootsuite.com/facebook-algorithm/ Accessed 12 Dec 2021.
2. Sehl K, Tien S. 6 ways to calculate engagement rate. June 29, 2021. https://blog.hootsuite.com/calculate-engagement-rate/ Accessed 12 Dec 2021.
